# The Sequelæ of Enteric Fever—I

**Published:** 1908-06-06

**Authors:** 


### Medicine.

### THE SEQUELÆ OF ENTERIC FEVER—I.

It is always difficult to draw any sharp line between those affections which arise during the course of a specific fever—its *complications*, and those other troubles which develop when the fever itself is past—its *sequelæ*. Nevertheless, there are certain affections, such as intestinal hæmorrhage, perforation of the bowel, parotitis, and the like, which are clearly complications of typhoid fever rather than sequelæ, whereas there are other conditions which, whether or not they start whilst there is still fever, arise or are still present when it has gone, and which, therefore, for purposes of discussion, may be grouped together as sequelæ. The great majority of all cases of typhoid fever that recover do so without any other sequela than prolonged and slow convalescence.

### Slow Convalescence.

Convalescence from typhoid fever is almost invariably a slow business. This is in marked contrast to some other severe febrile illnesses—lobar pneumonia, for example, after which a patient may often be back at work feeling perfectly well in something less than a month. Probably the durations of the respective pyrexias have something to do with this. Duration of fever, however, is by no means the only factor upon which the rapidity of convalescence depends, for influenza is a febrile illness in which the duration of pyrexia is much shorter even than that of lobar pneumonia, and yet it is often several weeks before the sufferer from influenza feels really well again. There is something about the influenza bacillus or its toxins which interferes with the recuperative powers of the tissue cells—particularly those of the nervous system—to a much greater extent than do pneumococci and their toxins. In this respect, the bacillus typhosus is comparable with the influenza bacillus rather than with the pneumococcus. In few cases is the patient anything like himself again in less than six weeks or two months; in the majority the time required is nearer six months than three; in some it may be a year before recovery is complete; whilst in an occasional case, fortunately rare, the patient never becomes quite his old self again.

There is one very marked difference between the convalescence of influenza and that of typhoid fever, and that is in their terminations. The convalescence of influenza again and again ends *by crisis*; for a variable number of days or weeks the patient, though back at work, feels that he is below the mark; suddenly, one day, he wakes up, and knows that he is feeling altogether a new man, entirely different to what he had been like the day before. This termination of the convalescence of influenza by crisis is quite remarkable; nothing like it can be observed after typhoid fever. Convalescence from the latter is *by lysis*, and is often spread over many weeks, or even months.

The importance of this from the point of view of the patient's business is obvious. Many patients are obliged to return to work a month or six weeks after fever has ceased; they are only just fit to do so, and they are obliged by circumstances to court the development of neurasthenia. Those who are in better circumstances should not attempt to return to business until they have had a long change of scenery and air, an average time to recommend being three or four months from the time when they were first placed upon full diet. Long before this they may say that they are as strong and well as ever they were, but they say this when they are holiday-making, and are not engaged in brain-work amongst business matters and worries. Muscular power recovers more quickly than nervous power after typhoid fever, and it is when an occupation entails brain work and worry that a return to it after the illness should be longest postponed.

### Œdema of the Legs.

It is not at all uncommon for a patient who is well on the road to recovery from typhoid fever to suffer from troublesome swelling of the ankles when the feet are allowed down and walking is first attempted. This swelling is of quite a different order to that which accompanies venous thrombosis; it is unassociated with pyrexia or pain; it is symmetrical; it seldom reaches far up the calf; it disappears entirely when the patient has been a night in bed, to return when the legs are put down again next day. It is not accompanied by albuminuria or any nephritic symptoms. It is, indeed, nothing more than a mal-nutritional œdema, occurring in the tissues furthest from the heart when they have been for a long time subjected to a wasting febrile process, and when they have also been long in the horizontal position without daily variations in their vasomotor requirements or in the quantity of lymph needed for their nourishment. Anæmia after the illness is an additional factor; similar œdema is of frequent occurrence in all sorts of cachexias.

This œdema is not more common in the old than the young, nor in females than males. Even schoolboys who were in the best of health until they contracted enteric fever may develop post-typhoidal œdema. It seldom persists; as the general health and strength improve, and the nutrition of the whole body, including that of the legs, approaches nearer and nearer to normal, the swelling becomes less and less marked, until finally it ceases altogether.

If one thing predisposes to this œdema more than another it is, probably, being in too great haste to get the feet to the ground. The recovery of tone and of adaptability in the vascular, vasomotor, and lymphatic systems in the legs takes just as long as does the restoration of the tone of the body generally. It is wise, therefore, to have the feet to the ground at first for only very short periods, repeated at intervals during the day, rather than for a longer period consecutively; the circulatory phenomena in the legs will thus become reaccustomed to variations in posture, and presently, by first bearing the weight upon the feet, then walking for a few minutes at a time, and so on day by day, complete restoration of leg power will come about without any œdema.

(*To be continued.*)

